# Recurrence-Free Survival in Composite Hemangioendothelioma: A Case Study and Updated Systematic Review

**DOI:** 10.3390/jcm14082541

**Published:** 2025-04-08

**Authors:** Milorad Reljic, Nina Rajovic, Jelena Rakocevic, Boris Tadic, Ksenija Markovic, Slavenko Ostojic, Milos Raspopovic, Borislav Toskovic, Jelena Vladicic Masic, Srdjan Masic, Natasa Milic, Djordje Knezevic

**Affiliations:** 1 Department for HBP Surgery, Clinic for Digestive Surgery, University Clinical Centre of Serbia, 11000 Belgrade, Serbia; 2 Institute for Medical Statistics and Informatics, Faculty of Medicine University of Belgrade, 11000 Belgrade, Serbia; 3 Institute of Histology and Embryology “Aleksandar Đ. Kostić”, Faculty of Medicine University of Belgrade, 11000 Belgrade, Serbia; 4 Department for Surgery with Anesthesiology, Faculty of Medicine University of Belgrade, 11000 Belgrade, Serbia; 5 Clinic of Emergency Surgery, University Clinical Center of Serbia, 11000 Belgrade, Serbia; 6 Department of Internal Medicine, Faculty of Medicine Foca University of East Sarajevo, 73300 Foca, Bosnia and Herzegovina; 7 Department for Primary Health Care and Public Health, Faculty of Medicine Foca University of East Sarajevo, 73300 Foca, Bosnia and Herzegovina

**Keywords:** composite hemangioendothelioma, systematic review, recurrence

## Abstract

**Background/Objectives**: Composite hemangioendothelioma (CHE) is a rare vascular endothelial tumor with borderline malignancy. This study presents a case of CHE and an updated systematic review of previously reported cases, providing insights into recurrence patterns and survival outcomes. **Methods**: A comprehensive electronic search was conducted across PubMed, Scopus, the Cochrane Library, and Web of Science up to 31 December 2024, to identify eligible case reports. Kaplan–Meier curves were used to estimate event-free survival. **Results**: We report a 61-year-old man with a splenic lesion associated with weight loss and abdominal pain persisting for 1 year. Intraoperative findings revealed an enlarged spleen and multiple hepatic deposits. Splenectomy and liver biopsy revealed a well-demarcated, nodular tumor measuring 160 × 145 × 100 mm, with histological and immunohistochemical findings consistent with CHE, complicated by hepatic metastasis. Of 405 potentially eligible studies, 59 were included in the review, covering cases from 2000 to 2024, with a peak in 2020 and 2023. The median age of patients was 42 years, with the most common tumor sites being the lower extremities (30.48%), followed by the face, head, and neck (20.95%), and upper extremities (18.1%). Surgical intervention was the most common treatment (60.95%). Recurrence-free survival was observed in 42.86% of cases, while 15.24% experienced recurrence with or without metastasis. Two patients (1.90%) died from the disease. The median recurrence-free survival was 48 months (95% CI: 7.3–88.7). **Conclusions**: CHE exhibits significant morphological variation and can mimic other vascular tumors. Accurate diagnosis is crucial for proper prognosis and avoiding overtreatment due to misdiagnosis as more aggressive neoplasms. Patients with high-risk CHE should undergo closer surveillance to ensure timely detection of progression.

## 1. Introduction

Composite hemangioendothelioma (CHE) is a highly uncommon vascular endothelial tumor with borderline malignancy, initially documented by Nayler et al. [1]. It was included in the World Health Organization (WHO) classification of Tumors of Soft Tissue and Bone in 2002, where it was defined as a “locally aggressive neoplasm with vascular differentiation that infrequently metastasizes, comprising a mixture of histologically benign, intermediate, and malignant components” [2]. In the revised 2013 WHO classification, the definition was updated to describe the tumor as a “locally aggressive, rarely metastasizing vascular neoplasm, composed of an admixture of histologically distinct components” [3]. This revision shifted focus to the presence of distinct histological components rather than categorizing them as benign, intermediate, or malignant [4].

The main characteristic of CHE is the presence of different vascular components, such as epithelioid hemangioendothelioma (EHE), retiform hemangioendothelioma (RHE), low grade angiosarcoma, lymphangioma, and different forms of hemangioma (capillary, cavernous, spindle cell, hobnail, etc). The histopathologic diagnosis of CHE necessitates the identification of at least two distinct components. The epithelioid and retiform variants are the most frequently observed, with angiosarcoma-like areas being commonly detected within the tumor [5].

Since CHE could contain angiosarcoma-line areas, it may be misdiagnosed as angiosarcoma. However, CHE typically features well-formed blood vessels, capillary structures, and hypercellular areas containing benign, intermediate, and malignant elements. In contrast, angiosarcoma is marked by malignant vascular structures, pleomorphic, atypical cells, and disorganized blood vessels [6]. Both CHE and angiosarcoma show immunopositivity for CD31, CD34, and VEGF, but angiosarcoma tends to exhibit more diffuse staining, while CHE demonstrates a more heterogeneous staining pattern. Therefore, a thorough histopathological evaluation is essential to distinguish the less aggressive CHE, which has a more favorable prognosis, from the highly malignant angiosarcoma, which carries a poorer prognosis [7].

CHE shares some molecular and genetic features with other vascular neoplasms. However, genetic alterations observed in CHE are relatively mild compared to more aggressive tumors, such as angiosarcoma. Cases of CHE are characterized by heterogeneous genetic and molecular profiles. However, certain specific gene fusions are more frequently observed in CHE than in other vascular tumors, which may contribute to their differential diagnosis from more aggressive neoplasms. The most characteristic are YAP1-FAM118B fusion YAP1-MAML2 fusion, including genes important for endothelial cell differentiation, cell growth, proliferation, and apoptosis, resulting in oncogenic alteration and promoting cell proliferation, survival, and angiogenesis [8].

CHE has more favorable biological behavior than angiosarcoma, even in cases where angiosarcoma-like areas are present. CHEs are currently classified as borderline neoplasms due to their propensity for local recurrence and infrequent metastasis. Research indicates that local recurrence occurs in approximately 57% of cases, with timelines ranging from 18 months to 10 years after the initial surgical excision [9]. Consequently, wide local surgical excision is recommended as the primary treatment strategy [10,11]. This relatively high recurrence rate underscores the necessity for diligent postoperative monitoring.

However, a significant gap remains regarding the precise timeline for tumor recurrence. To address this gap, we present a case of CHE treated at our clinical center, accompanied by an updated systematic review of previously reported cases. This review includes an analysis of the recurrence-free survival across all documented cases, providing valuable insights into the tumor’s recurrence patterns and contributing to the existing body of knowledge on CHE.

## 2. Materials and Methods

The systematic review was undertaken in accordance with Preferred Reporting Items for Systematic Reviews and Meta-Analyses (PRISMA) [12]. The review methods were predefined, and no significant deviations from the established protocol were observed.

### 2.1. Study Selection

The screening process for study inclusion in the systematic review was conducted in two phases. At each stage, two reviewers independently assessed the studies, resolving disagreements through discussion or consensus. A third reviewer was consulted to resolve any remaining discrepancies. Eligible studies included published case reports on composite hemangioendothelioma. Exclusion criteria were (1) examined populations other than those with CHE (EHE, RHE, etc.), (2) absence of relevant outcomes, (3) studies not classified as case reports or case series (reviews), (4) wrong population (not humans), and (5) publications not in the English language. 

### 2.2. Search Strategy

The search strategy was collaboratively developed by two reviewers, one with expertise in general surgery and the other with experience in search strategy design. A comprehensive electronic search of PubMed, Scopus, the Cochrane Library, and Web of Science was conducted up to 31 December 2024, to identify published studies containing the keyword “composite hemangioendothelioma”.

### 2.3. Data Abstraction

The following data were independently extracted by two reviewers: study title, author(s), year of publication, patient gender and age, tumor location, multifocality, tumor size, symptom duration, treatment approach, histological components, evidence of recurrence, and follow-up duration.

### 2.4. Data Analysis

Data are expressed as median (min–max) or n (%). Kaplan–Meier curve and Log rank test were used to estimate event-free survival. Disease recurrence was defined as either local recurrence or the development of new metastatic disease. Only cases with available data on this outcome were included in the analysis. A *p* value of <0.05 was considered to be statistically significant. Analysis was performed using SPSS for Windows (21.0; IBM SPSS, Chicago, IL, USA).

## 3. Results

### 3.1. Case Presentation

The Hematology Council at the Clinic for Hematology, University Clinical Center of Serbia, referred a 61-year-old male for further evaluation in February 2024. A splenic lesion of uncertain nature, associated with weight loss and abdominal pain persisting for one year, prompted the referral. On admission, the patient was afebrile, with a blood pressure of 130/85 mmHg, a heart rate of 75 bpm, and SpO_2_ of 98%. The patient denied any chronic illness, comorbidities, or surgical history. Additionally, the patient denied tobacco use and drank alcohol only on social occasions. The patient’s family history was negative for any malignancies. Abdominal examination revealed a soft abdomen with mild tenderness in the upper quadrants. On initial laboratory analysis, white blood cell count and C-reactive protein were elevated, while hemoglobin levels and red blood cell count were slightly below normal range. The patient had quite hypoalbuminemia. The numbers of platelets and tumor markers (alpha-fetoprotein, carcinoembryonic antigen, and carbohydrate antigen 19-9) were all within the reference range (Table 1).

**Figure 1:**
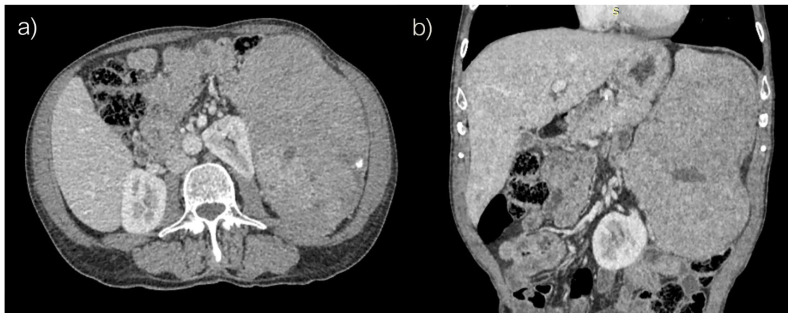
Abdominal computed tomography, (**a**) axial and (**b**) coronar scans.

Computed tomography revealed voluminous spleen, an inhomogeneous parenchymal structure with an extensive heterodense lesion with irregular hypodense zones and a post-contrast viable solid component. Peripheral smaller punctiform nodular calcifications were also detected (Figure 1).

Intraoperative findings confirmed an enlarged spleen, with multiple hepatic deposits identified. Subsequently, splenectomy and liver biopsy were performed. A clearly demarcated, nodular tumor measuring 160 × 145 × 100 mm was observed. The tumor was predominantly solid (70–80%), with a structureless to granular appearance, while the remaining portion (20–30%) was pseudocystic, containing cavities up to 30 mm in size, some filled with gelatinous, zigzag-patterned contents and others with a bloody appearance. The solid portion of the tumor revealed areas of hyalinization, the largest of which measured 45 × 40 × 35 mm, alongside focal necrotic areas, which constituted approximately 10% of the lesion’s height. The surrounding splenic parenchyma appeared dark red and fatty, with small, accentuated nodules of white pulp measuring up to 2 mm in diameter. No lymph nodes were observed within the sparse fatty tissue of the hilum.

**Figure 2:**
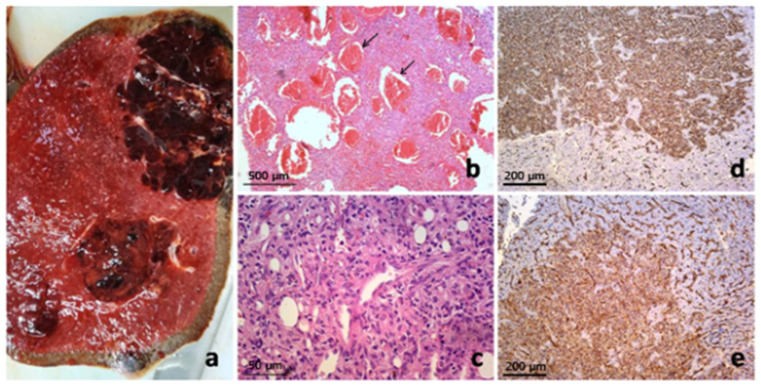
Pathology of composite hemangioendothelioma of the spleen with hepatic metastases: Macroscopic presentation of primary splenic vascular tumor on cross-section (**a**) and pathohistological finding on low magnification simulated architecture of cavernous hemangioma (arrows) (**b**), but on closer inspection revealed admixture of several vasoformative patterns with more cellular areas of epitheloid and spindled endothelial cells (**c**). Metastatic liver deposits showed the same histomorphology and clear CD34 (**d**) and CD31 immunoexpression (stained brown) (**e**).

Immunohistochemical analysis of the tumor cells showed strong immunoreactivity for vimentin (++), CD31 (++), and ERG (++), with most tumor cells also positive for CD68 (++). Focal or inhomogeneous positivity for actin SMA-alpha (+/++) was noted, while only a small number of tumor cells showed positivity for LCA, corresponding to the inflammatory infiltrate. No immunoreactivity was observed for CD34, S-100 protein, pancytokeratin AE1/AE3, or EMA. The immunophenotype observed was consistent with a splenic vascular tumor, specifically “littoral cell angioma”; however, its morphology and biological behavior differed significantly. The proliferative index, measured by Ki-67, was approximately 25%. Histomorphological and immunohistochemical findings were most consistent with a diagnosis of “Composite Hemangioendothelioma (CHE), with hepatic metastasis” (Figure 2).

On the second postoperative day, after the start of the oral diet, the abdominal drainage began to drain about 300 mL of yellowish-white, milky fluid. The fluid was subjected to biochemical analysis and tested positive for triglycerides, so it was found to be chylous ascites. The patient was treated with a low-fat, low-salt diet and somatostatin analogs. The amount in the drainage decreased significantly within 5 days. Seven days after the operation, the control ultrasound examination of the abdomen showed normal postoperative findings without fluid accumulation in the abdominal cavity. The abdominal drainage remained in situ for eight days. The patient was subsequently referred to the oncology council, where symptomatic and supportive therapy was recommended due to the progressive deterioration of the overall condition. The patient died due to advanced metastatic disease six months later.

### 3.2. Systematic Review

**Figure 3:**
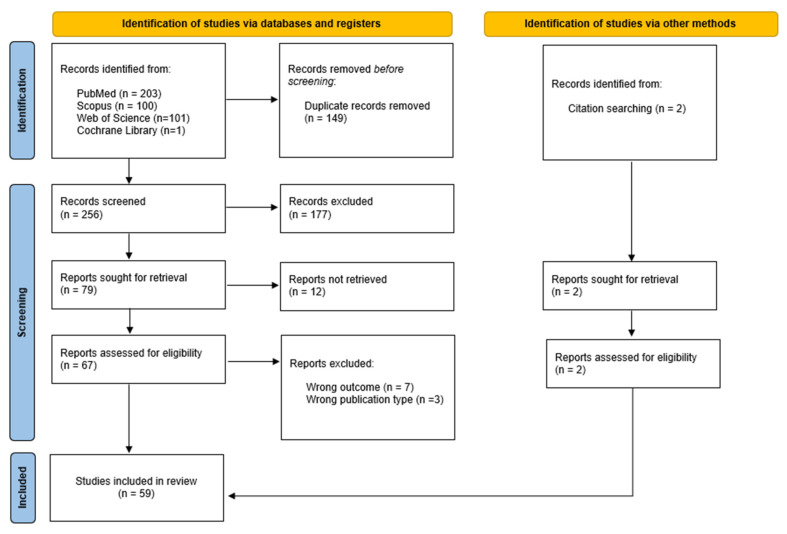
Flowchart of study selection process.

A total of 405 potentially eligible case reports were identified across four electronic databases. After removing duplicates, 256 titles and abstracts were screened for relevance. Of these, 177 reports did not meet the eligibility criteria, leaving 79 case reports for further assessment. Twelve reports could not be retrieved, resulting in sixty-seven reports undergoing full-text evaluation. Following this screening, seven reports were excluded due to irrelevant outcomes and three due to incorrect publication type. Additionally, two relevant case reports were identified during the full-text review and included in the final review. In total, 59 studies were selected for review. The study selection process is detailed in Figure 3, following the PRISMA flow diagram.

### 3.3. Characteristics of Eligible Case Reports

**Figure 4:**
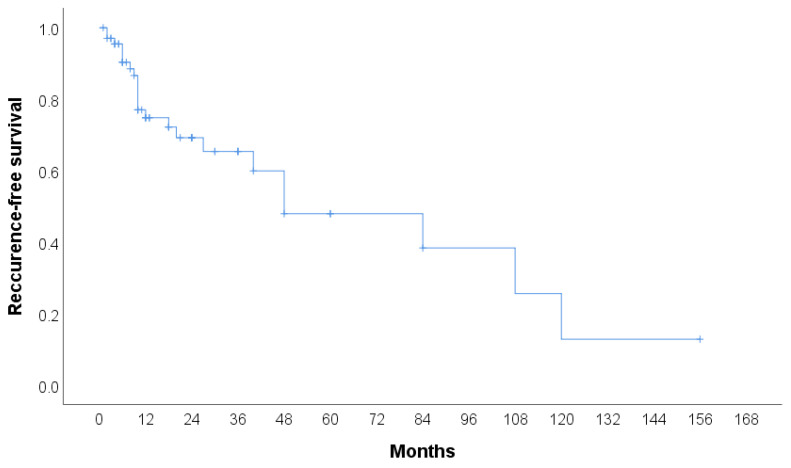
Kaplan–Meier curve for recurrence-free survival.

**Figure 5:**
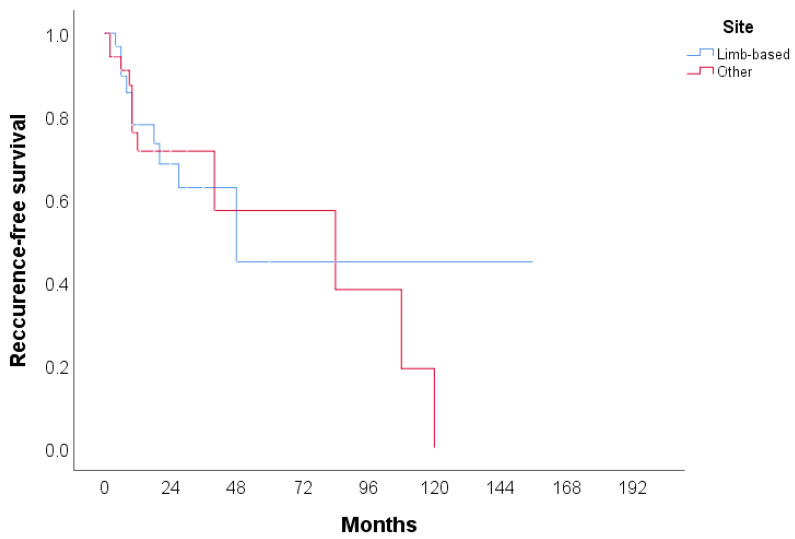
Kaplan–Meier curve for recurrence-free survival according to anatomical site.

The clinical features of CHE reported in the literature (n = 106) are summarized in Table 2. A total of 105 cases were identified, with more than half of the patients being female (53.3%). The median age of the patients was 42 years, with the youngest patient being 9 months old and the oldest 80 years old. The most common tumor site was the lower extremities (including the hip) (30.48%), followed by the face, head, and neck (20.95%), and the upper extremities (including the brachial plexus) (18.1%). The most frequently used treatment modality was surgery (60.95%), followed by surgery with radiotherapy (6.67%) and surgery with chemoradiotherapy (4.76%). In 42.86% of cases, no recurrence was observed, while 15.24% experienced local recurrence, either alone or in combination with metastasis. Additionally, two patients (1.90%) died due to the disease. The cases included in the review span from 2000 to 2024, with the majority of cases reported in 2020 and 2023. Detailed characteristics of all 59 publications included in the systematic review, including the present case, are provided in Table 3, Table 4 and Table 5. Recurrence-free survival is presented in Figure 4. A total of 68 cases with 23 events were used in analysis. Median recurrence-free survival was 48 months (95% CI: 7.3–88.7). Kaplan–Meier curve for recurrence-free survival according to the anatomical site is presented in Figure 5. No significant difference was found in recurrence-free survival between limb-based CHE and CHE localized on other anatomical sites (*p* = 0.700)

## 4. Discussion

CHE is an exceedingly rare neoplasm, with less than 100 cases documented in the English literature to date [61]. The true incidence of CHE may be underreported due to its histological similarity to a diverse array of other vascular neoplasms. The rates of recurrence of CHEs are shown to be relatively high, with rare occurrences of regional lymph node metastasis, while distant metastases remain exceptionally uncommon. This systematic review updates the current number of reported cases of CHE and identifies a median recurrence time of 48 months. These findings underscore the importance of ongoing surveillance of these patients, particularly during the initial years following diagnosis.

Among the 105 documented cases, the age of CHE onset ranges from 9 months [54] to 80 years [47], with a median age of 42 years. Patients with CHE exhibited a female predominance (56/105) and a site preference for the extremities (51/105). Patients usually presented with a prolonged history of lesions, ranging from several months to several years prior to diagnosis. Similarly, in our patient symptoms persisted for one year, and ultimately, the present case involved a primary tumor in the spleen with multiple metastases, a condition that has been documented in only two other cases to date (25,41). In both of those cases, patients presented with splenomegaly due to the presence of a primary tumor, with multiple liver metastases. However, in one case the patient was asymptomatic despite the extensive disease; lesions were not only located in the spleen and liver, but also in the lungs with multiple bone metastases [41]. A case presented by Yoda et al. [25] had similar clinical characteristics as our patient, presenting with abdominal pain, primary splenic lesion, and metastases in the liver.

The exact etiology of CHE remains largely unknown; however, these tumors are often associated with underlying vascular abnormalities. Many CHEs contain benign vascular components, such as spindle-cell HE, and in some instances, angiomatosis [65]. Other vascular entities, including arteriovenous malformation, epithelioid HE, retiform HE, papillary intralymphatic angioendothelioma, and kaposiform HE, have also been observed in these tumors. These findings suggest that some cases of CHE may, at least in part, arise from a malformational origin, given the presence of these benign vascular elements. In comparison to conventional angiosarcoma which is typically more aggressive, most CHEs exhibit less aggressive behavior [65]. A review of the existing literature reveals that the most common histological pattern observed in CHE is retiform HE, followed by spindle-cell and epithelioid HE. Previous cases of primary splenic CHE were predominantly composed of spindle cell HE, retiform HE, and epithelioid HE [25], as well as epithelioid HE, papillary intralymphatic angioendothelioma (PILA), and hemangioma-like areas [41], which is similar to pathohistological findings in our presented case.

CHE is characterized by the presence of a combination of different vascular components. Therefore, careful pathohistological analysis is of utmost importance for the precise diagnosis of CHE. Heterogenous pathohistological patterns within CHE exhibit different malignant potential. This may explain the possibility of immunohistochemical patterns differing from one case to the other, therefore perplexing the correct CHE diagnosis. The most common finding in cases of CHE is immunopositivity of vascular endothelial markers, CD34 and CD31, followed by ERG immunopositivity. Tumor cells in our presented case show strong immunoreactivity for CD31 and ERG, similarly to the case presented by Li et al. [41], but with no CD34 immunopositivity. Immunohistochemical profile of tumor cells in our patient showed vimentin, CD68, and alpha-SMA positivity. However, these markers were not tested in previous cases of splenic CHE, which constrains detailed immunohistochemical comparison and profiling.

Reviewing the literature on CHE cases with available immunoexpression data for CD31, CD34, and ERG, we found that 17 out of 25 (68%) cases were positive for all three markers (Table 4), a pattern also seen in our presented case. In the remaining eight cases (32%) where triple positivity for CD31, CD34, and ERG was not observed, all lacked CD34 expression but showed immunopositivity for both CD31 and ERG.

Given the complexity and unique pathological characteristics of CHE, most previously published cases have primarily focused on histopathology and immunohistochemical findings, while reports detailing its imaging features remain limited. Deng et al. [54] state that CHE shares common features on CT and MRI. Due to its hypervascular nature, it often appears hyperintense on T2-weighted MRI and shows strong contrast enhancement. As an intermediate-grade vascular tumor, CHE tends to grow slowly, often displaying well-defined margins with varying degrees of sclerosis, helping to differentiate it from more aggressive malignancies. The authors conclude that CT and MRI play a crucial role in defining tumor margins and guiding both preoperative and postoperative treatment planning. However, Deng. and colleagues point out that bone scans lack specificity in CHE, and therefore, MRI and CT should be the primary imaging modalities, with bone scans used for screening multicentric disease. PET-CT, though rarely reported in CHE, can help assess malignancy due to its ability to detect increased FDG uptake, which suggests aggressive tumor behavior. It also provides systemic functional imaging, making it useful for staging and follow-up.

Circulating tumor DNA (ctDNA) and novel biomarkers have emerged as promising tools for assessing tumor burden, monitoring treatment response, and predicting recurrence risk in various malignancies [66]. Presently, the literature does not document any clinically significant or distinctive molecular or genetic discoveries in CHE. The molecular findings in composite hemangioendotheliomas align with the characteristic molecular findings of their individual histologic patterns [67]. Antonescu et al. [8] stated that no recurrent genetic abnormalities have been documented to date in either RHE or CHE, although Perry et al. identified one instance of PTBP1-MAML2 translocation and one instance of EPC1-PHC2 fusion transcripts in patients with CHE. It is important to note that Perry et al. [4] come to the conclusion that this unusual hemangioendothelioma with neuroendocrine features most likely represents a clinically aggressive variant of CHE. However, they also raise the possibility that this hemangioendothelioma could be an unusual variant of RHE or even an entirely different entity. If these gene fusions are consistently found to be associated with the aggressive clinical behavior of CHE, they may serve as valuable molecular biomarkers for risk stratification and disease monitoring. Their identification in routine clinical practice could facilitate early detection of high-risk cases, guide treatment decisions, and improve patient management, as well as prediction of recurrence risk. Further research is necessary to validate their prognostic significance and determine their potential role in personalized therapeutic strategies.

Although surgical excision is frequently curative for CHE, local recurrence—sometimes aggressive—has been reported in the literature. These updated findings provide insights regarding the median recurrence time, contributing to a more accurate understanding of the disease’s progression. The relatively high incidence of local recurrence in CHE can be attributed to several factors that complicate effective surgical management. A key factor is the multicentric nature of the neoplasm, where the tumor may originate from multiple sites within the affected organ. This multifocality complicates the identification and complete excision of all neoplastic tissue, increasing the difficulty of achieving a clear surgical margin. In cases where tumor margins are not clearly delineated, the likelihood of residual disease is increased, thereby augmenting the potential for recurrence. However, there is insufficient evidence in the existing literature to conclusively determine whether the use of aggressive surgical margins leads to improved recurrence-free survival. Further research is needed to clarify the impact of surgical margin width on long-term patient outcomes. Additionally, adjuvant therapy may be necessary in cases of subtotal resection due to the risk of local recurrence and potential for malignancy. A subtotal resection or biopsy may not provide a comprehensive representation of the entire neoplasm, suggesting that CHEs may be underreported in the literature [67]. According to the present study, median time to recurrence was found to be 48 months; this can be of immense importance for multidisciplinary teams, as collaboration between surgeons and the histopathology department, for achieving optimal outcomes. Thorough preoperative imaging and meticulous surgical planning are the key strategies to minimize the risk of residual disease and improve long-term patient prognosis.

Based on the available data, the impact of different treatment modalities on patient outcomes varies. The majority of patients underwent surgery alone; however, their outcomes were inconsistent. Among the five patients who received surgery combined with radiotherapy and had available outcome data, all remained recurrence-free. In contrast, among the four patients treated with surgery and chemotherapy with available outcome data, two experienced recurrences, one remained alive with disease, and one remained recurrence-free. The single patient who underwent a combination of surgery, radiotherapy, and chemotherapy developed a relapse with lung metastasis. These findings suggest that while surgery alone may lead to favorable outcomes in some cases, the addition of radiotherapy or chemotherapy does not consistently prevent relapse or metastasis. However, the small sample size and limited long-term follow-up data restrict the ability to draw definitive conclusions regarding the efficacy of these treatment approaches. Additionally, the study is subject to reporting bias, as retrospective data collection may lead to underreporting or inconsistent documentation of treatment responses and outcomes. Another limitation is the lack of available data on newer therapeutic approaches, such as vascular endothelial growth factor (VEGF)-targeted therapies, which have shown promise in the treatment of other vascular tumors. However, the rarity of this disease hampers the development of clinical practice guidelines and the implementation of treatment recommendations.

*Recommendations*: Patients with high-risk CHE, including those with deep, infiltrative tumors, multifocal disease, local recurrence, or metastases, should undergo closer surveillance to ensure timely detection of progression [67]. For patients who had complete wide local excision, follow-up can be less frequent, as recurrence is rare. An initial follow-up at six months post-surgery is recommended to assess healing and recurrence, with subsequent visits as needed. However, for those with high-risk features, a more rigorous follow-up schedule is necessary. Patients on systemic therapy should undergo regular imaging and monthly follow-ups until tumor regression is confirmed. Once remission is achieved, follow-up intervals may be extended, but continued long-term monitoring remains essential. Given the potential for recurrence or progression, high-risk CHE patients should receive strict and frequent follow-up, tailored to their individual clinical course and treatment response.

## 5. Conclusions

CHE displays considerable morphological variation at low magnification, both within individual tumors and across tumors from different patients. It can mimic other vascular tumors, both clinically and histologically. Accurate diagnosis is essential due to the potential for recurrence of CHE. This is important not only for precise prognostication but also to avoid unnecessary overtreatment that may arise from misidentification as more aggressive neoplasms, such as angiosarcoma and epithelioid HE. However, patients with high-risk CHE should undergo closer surveillance to ensure timely detection of progression. While surgery alone may lead to favorable outcomes, the small sample size restricts the ability to draw definitive conclusions regarding the efficacy of adjuvant chemotherapy or radiotherapy in patients with CHE.

## Figures and Tables

**Table 1:** Initial laboratory tests results.

Laboratory Test	Result	Reference Range
White blood cells	14.4	3.4–9.7 × 10^9^/L
Hemoglobin	118	138–175 g/L
Red blood cells	4.15	4.34–5.72 × 10^12^/L
Platelet count	312	58–424 × 10^9^/L
C-reactive protein	32.4	0.0–8.0 mg/L
Albumine	22	34–55 g/L
Alpha-fetoprotein	2.3	1.1–8.0 μg/L
Carcinoembryonic antigen	<1.73	0.0–5.0 μg/L
Carbohydrate antigen 19–9	<2.06	0–37 U/mL

**Table 2:** Clinical features of CHE (n = 105) reported in the literature.

Variable	n (%)
**Gender**	
Male	49 (46.7)
Female	56 (53.3)
Age (yrs), median (min *–max)	42 (9 mo–80 yrs)
**Site**	
Lower extremities (incl. hip)	32 (30.48)
Upper extremities (incl. brachial plexus)	19 (18.1)
Face, head and neck	22 (20.95)
Back	2 (1.9)
Mediastinum (incl. mediastinal organs)	8 (7.62)
Abdominal organs	8 (7.62)
Thorax	2 (1.9)
Abdominal wall and cavity	4 (3.81)
Vertebral and paravertebral region (incl. spinal cord)	6 (6.71)
Pelvis (incl. anogenital region)	2 (1.9)
**Treatment**	
Surgery	61 (57.5)
Surgery and radiotherapy	8 (7.5)
Surgery and chemoradiotherapy	6 (5.7)
Surgery and radiotherapy and chemoradiotherapy	1 (0.9)
Biopsy	4 (3.81)
Electron beam	1 (0.95)
Radiation	1 (0.95)
Chemotherapy	2 (1.95)
Radiotherapy and chemotherapy	1 (0.95)
Not reported	21 (20.0)
**Outcome**	
NSR	45 (42.86)
LR/Met/LR and Met	17 (15.24)
AWOD	3 (2.86)
AWD	7 (6.67)
DOD	1 (1.90)
Not available	32 (30.48)

* for analysis, 9 months = 1 y; mo, months; yrs, years; incl, including; NSR, no sign of recurrence; LR, local recurrence; Met, metastases; AWOD, alive without disease; AWD, alive with disease; DOD, dead of disease.

**Table 3:** Characteristics of studies included in the systematic review.

Author	Year	Sex	Age	Site	Multi-Focal	Largest Diameter * (cm)	Duration (Months)	Treatment	Histological Components	Outcome (Follow Up)
Nayler et al. [1]	2000	M	42	Dorsum right foot	N	6 × 4.5 × 4	12 yrs	Surgery, radiotherapy or excision of recurrences	EHE, SCHE, AS-like	NSR (1 yrs)
		F	27	Dorsum right foot	N	4 nodules ranging from 0.7 to 2	Since childhood	Surgery	EHE, RHE, SCHE, AS-like	RR (4 yrs) followed by below-knee amputation; NSR (6 yrs later)
		M	21	Left index finger	N	2 nodules of unstated size	Several mo	Surgery	RHE, SCHE, AV malformation	NSR (13 yrs)
		M	44	Left third finger	N	1	Several yrs	Surgery	EHE, RHE, AS-like	NSR (2 yrs)
		M	70	Tongue	N	/	/	Surgery	EHE, RHE, AS-like	Metastasis to submandibular node (9 yrs), RR (10 yrs), metastasis to thigh (11 yrs)
		F	31	Dorsum of foot	N	1	2 yrs	Surgery	EHE, RHE, SCHE, AS-like	/
		F	71	Dorsum of foot	N	3–4	6 yrs	Surgery	EHE, RHE, AS-like, Lymphangioma circumscriptum	/
		M	35	Dorsum of hand	N	3	Several yrs	Surgery	EHE, RHE, AS-like	RR (4 yrs)
Reis-Filho et al. [13]	2002	F	23	Forearm, hand	N	13 × 13 × 7	Since infancy	Surgery	RHE, SCHE, Cavernous hemangioma, EHE, AS-like	NSR or metastasis (7 yrs)
Sapunar et al. [14]	2003	M	43	Toe	/	/	/	Surgery, radiotherapy	/	/
Biagioli et al. [11]	2005	F	46	Toe	N	2.0 × 1.5	3yrs	Surgery	RHE, EHE, SCHE	First RR (2 yrs), second RR (18 mo), NSR (after 1 y)
Chu et al. [15]	2006	F	11	Axilla	N	6.0 × 4.5 × 4.0	2mo	Surgery	Cavernous HE, Capillary HE, RHE, AS, KHE, EHE, SCHE, AV malformation	Lymph node metastases at diagnosis, bone metastases (4 mo), lung, bones, and liver metastases (after 2 yrs), AWRD
Tronnier et al. [16]	2006	F	73	I: Third toe II: Between 1st and 2nd toe III: Foot IV: Achilles tendon	Y	2.8 × 3.1 × 1.0 2.0 × 1.9 × 1.4 / /	1.5 yrs 10 yrs Few mo Few mo	Surgery	- I: EHE - II: biopsy not performed - III: EHE - IV: SCHE	RR (20 mo)
Fasolis et al. [17]	2007	M	38	Oral cavity, cheek mucosa	N	2.5 × 2	/	Surgery	RHE, EHE, AS-like	NSR (30 mo)
Fukuanga et al. [18]	2007	F	39	Left lower thigh and foot	Y	30	Since birth	Surgery	EHE, RHE, SCHE, AS-like, Lymphangioma	AWD at 39 yrs
		M	44	Mandibular vestibule	N	1.3	4–6 mo	Surgery	EHE, RHE	NSR (13 mo)
		F	75	Lower thigh		3.5	10 yrs	Surgery	EHE, RHE, AS-like	RR (27 mo)
		F	37	Upper arm		4	Since birth	Surgery	EHE, RHE, AS-like, angiomatosis, AV malformation, Cavernous hemangioma	AWD since birth
		F	22	Foot		5	3 yrs	Surgery	EHE, RHE	/
Requena et al. † [9]	2007	M	60	Leg and foot	Y	/	Since childhood	Surgery, chemotherapy (interferonα2a and melphalan)	RHE, SCHE, EHE, PILA	RR and lymph node metastasis (within mo), AWD (1 yrs)
Tejera-Vaquerizo et al. [19]	2008	F	23	Back	N	3	2 yrs	Surgery	EHE, RHE, AS-like, SCHE	NSR (30 mo)
Utas et al. [20]	2008	F	62	Forearm and hand	N	5 × 9	4 mo	Chemoradiotherapy (interferon alpha 2b), surgery	SCHE, Cavernous hemangioma, Epitheloid hemangioma, RHE, AS-like	/
Aydingoz et al. † [21]	2009	F	48	Thigh	N	1–1.5	2 yrs	Surgery chemotherapy (epirubicin and iphosphamide)	Capillary hemangioma, EHE, SCHE, Kaposi sarcoma-like	Multiple RR (within mo), lymph node metastasis (2 yrs), NSR (2 yrs)
Cakir et al. [22]	2009	F	50	Mediastinum	N	6x4x3	2 mo	Surgery	RHE, Epithelioid-like HE, Kaposiform HE-like, Spindle cell hemangioma, AS-like	NSR (13 mo)
Tsai et al. [23]	2011	F	23	Foot	N	4	5 yrs	Surgery	EHE, RHE	NSR or metastasis (7 mo)
		F	15	Hypopharynx	N	3.2	3 mo	Surgery	SCHE, AS-like	NSR or metastasis (18 mo)
		F	59	Hypopharynx	N	2.4	2 mo	Surgery	SCHE, EHE, RHE	NSR or metastasis (10 mo)
		M	8	Elbow	N	1.6	18 mo	Surgery	SCHE, RHE	NSR or metastasis (48 mo)
Chen et al. [24]	2012	F	46	Neck	N	4.8	4 yrs	Surgery	EHE, RHE, PILA	/
Yoda et al. [25]	2012	F	67	Spleen	N	/	4 mo	Surgery chemotherapy (paclitaxel)	SCHE, RHE, EHE	/
Liau et al. [26]	2013	F	24	Scalp	N	1.5	Several mo	Surgery	EHE, RHE, Dabska tumor-like, AS-like	NSR (1 yrs)
McNab et al. [27]	2013	M	75	Knee	N	/	/	Biopsy	SCHE, Kaposi sarcoma-like, AS-like	RR, increased pain at tumor site, (3 mo after chemotherapy with Taxol)
Tateishi et al. [28]	2013	F	34	Nose	N	0.8 × 0.8	7 mo	Electron beam	RHE, EHE	NSR (9 mo)
Zhang et al. [29]	2013	F	32	Kidney	N	2.6 × 2.1	1 week	Surgery	AS-like, EHE, SCHE	NSR or metastasis (11 mo)
Dong et al. [10]	2014	M	56	Manubrium Sterni	/	/	2 yrs	Surgery	N/A	RR (10 yrs)
Mahmoudizad et al. [5]	2014	M	68	Vertex of scalp	N	6.3 × 5.3	10	Radiation	EHE, RHE, SCHE	/
Stojsic et al. [30]	2014	M	58	Gluteal region	N	2.7	Several years	Surgery	RHE, Sinusoidal hemangioma, AV hemangioma, AS-like	NSR (3 mo)
Leen et al. [31]	2015	M	43	Submandibular area	N	2.2	3 mo	Surgery	SCHE, RHE, EHE, AS-like	NSR (6 mo)
Bhat et al. [32]	2016	M	31	Upper back	N	1.7 × 1.6	1 yrs	Surgery	RHE, EHE, Cavernous angioma-like	NSR or metastasis (5 mo)
Perry et al. [4] º	2017	M	47	Wrist	/	7.7	/	/	/	RR, metastasis (liver, lung, humerus), DOD
		F	48	Right ankle	/		/	/	/	RR, AWOD
		F	36	Periaortic	/	2.1	/	/	Neuroendocrine-appearing and myoid-appearing cells	Metastasis (sacrum), AWD
		F	48	Vertebral	/	/	/	/	Nested HE and RHE, Hemangioma like vascular channels	Metastasis (lung), AWD
		M	27	Pulmonary vein	/	/	/	/	/	Metastasis (brain), AWD
		F	14	Ear	/	3.0	/	/	/	/
		F	55	Superficial hip	/	0.4	/	/	/	NSR
		M	55	Liver	/	6.9	/	/	/	NSR
		M	15	Foot	/	1.2	/	/	/	NSR
		F	59	Cheek	/	9.5	/	/	Retiform regions, Hemangioma-like region, Epithelioid regions	/
		M	9	Index finger	/	/	/	Surgery	Retiform areas, Solid nested areas	/
Rokni et al. [33]	2017	F	78	Face, scalp and eylids	Y	5 × 3 × 1	18	Surgery chemotherapy (thalidomide)	EHE, RHE	/
Sakamoto et al. [34]	2017	M	40	Leg, foot	Y	2–3	6 mo	Surgery radiation	EHE, SCHE	NSR or metastasis (2.5 yrs)
Umar et al. [35]	2017	M	9	Scalp, parietofrontal region	N	2 × 2 × 2	6 mo	Surgery	SCHE, PILA, RHE	NSR or metastasis (18 mo)
Harrington et al. [36]	2018	M	71	Lung	N	5.8	/	Surgery	- RHE	/
Antonescu et al. [8]	2020	F	9	Foot	/	/	/	/	RHE, EHE	/
		F	9	Heel	/	/	/	/	/	/
		F	7	Middle finger	/	/	/	/	/	/
		M	19	Hand	/	/	/	/	/	/
		F	56	Forearm	/	/	/	/	/	/
		F	24	Scalp	/	/	/	/	/	/
		F	36	Scalp	/	/	/	/	/	/
		M	35	Heel	/	/	/	/	/	/
		M	12	Shoulder	/	/	/	/	RHE, Hemangioma-like areas	/
		F	68	Buttock	/	/	/	/	/	/
		F	42	Finger	/	/	/	/	/	/
		M	37	Pancreas, liver, lung	Y			Liver biopsy	RHE, EHE	/
Chin et al. [37]	2020	F	67	Forearm	N	5 × 3	1 yrs	Surgery	AV malformation, SCHE, RHE, AS-like	NSR or metastasis (4 mo)
Cheuk et al. [38]	2020	F	53	Paravertrebral	N	5		Surgery	Epithelioid cells paraganglioma-like, SCHE, RHE, Cavernous hemangioma/lymphangioma, EHE	/
Gok et al. [39]	2020	M	54	Paravertrebral	N	3 × 2.5 × 2	2 yrs	Surgery	AS-like, Kaposi sarcoma-like, EHE, Retiform hemangioma	NSR or metastasis (1 yrs)
Langguth et al. [40]	2020	F	50	Pericardium	N	9.2 × 5.9 × 5.8		Surgery	EHE, Cavernous blood vessel configuration	/
Li et al. [41]	2020	M	65	Spleen	N	15.2	/	Surgery	EHE, PILA, Hemangioma-like	AWD (6 mo)
Liao et al. [42]	2020	F	28	Chest wall	N	1 × 1.2	20 yrs since childhood; new 3 mo ago	Surgery	EHE, RHE, AS-like, Lymphangioma	NSR or metastasis (8 mo)
Mani et al. [43]	2020	M	22	Distal femur (bones of the foot), tibia and pattela	Y	5.4	16	Surgery	EHE, SCHE, RHE	NSR (6 mo)
		M	36	Distant tibia, distant fibula and bones of the foot	Y	12.5	1	Surgery	EHE, RHE, SCHE	NSR (3 yrs)
Asilian et al. [44]	2021	F	30	Small right finger		0.6	Within few days following trauma	Surgery, chemoradiotherapy	RHE-like with papillary formation	NSR (6 mo)
Miyamoto et al. [45]	2021	M	71	Posterior chest wall, vertebras adjacent to the spinal canal	Y	10		Surgery, radiotherapy	EHE, RHE	NSR (12 mo)
Dermawan et al. [46]	2021	M	70	Lower nasopharynx, cervical lymph nodes	Y	2.5 and 2.8	3 yrs	Surgery, Radiotherapy, immunotherapy (ipilimumam + nivolumab)	EHE, AS-like	RR (2 mo), metastasis in lungs (1 yrs)
		F	71	Neck lymph node	N	2.8	/	Surgery	RHE, Cavernous hemangioma-like	NSR (3 mo)
Han et al. [47]	2022	F	38	Stomach	N	2	15 mo	Surgery	SCHE, EHE (multifocal micronodules)	NSR (18 mo)
Koutlas et al. [48]	2022	F	21	Mandibular vestibule	N	1	1 yrs	Biopsy	RHE, EHE, SCHE, AS morphology	NSR (24 mo)
Nakamura et al. [49]	2022	F	80	Cervical spine	Y	/	2 yrs	Surgery, radiotherapy	Kaposiform HE, AS (low grade)	NSR (21 mo)
Zhang et al. [50]	2022	M	46	Heart	N	1.3 × 1.4	/	Surgery	SCHE, EHE, EHE-like	NSR or metastasis (6 mo)
Zhou et al. [51]	2022	F	50	Heart	N	5.0 × 4.5 × 3.0	1 week	Surgery	EHE, Cavernous hemangioma	NSR (1 mo)
Balko et al. [52]	2023	M	49	Left lower extremity and gluteus	Y	43 × 78 mm 20 × 10 mm	/	Surgery	PILA, RHE, AS-like	NSR (2 yrs)
Bui et al. [53]	2023	M	40s	Penis	N	2.3 × 1.8 × 0.6	2 yrs	Surgery radiotherapy	Primary lesion: Retiform HE + epithelioid HE 1st recurrent lesion: Retiform HE with increase in cellularity and cytologic atypia; 2nd recurrent lesion: Reticular HE + epithelioid HE with high-grade features	NSR or metastasis (5 yrs)
Deng et al. [54]	2023	F	21	Thigh (left pubis)	N	3.5 × 3.6	2 yrs	Surgery	Kaposiform HE (dominant), RHE (dominant), SCHE, EHE	NSR or metastasis (5 yrs)
		M	66	Left ilium and T6, T12 vertebra	Y	3.0 × 2.6	6	Surgery	Cavernous HE, RHE, AS components	/
Dermawan et al. [55]	2023	F	70	Left hip involving abductor musculature	N	11.5	1 yrs	Surgery chemotherapy	Spindled and epithelioid cells forming solid sheets; UPS-like	AWD (metastasis to lung, 8 mo)
		M	71	Abdominal wall	Y	2.0	/	Chemotherapy (decitabine, docetaxel)	Malignant epithelioid neoplasm forming solid sheets	DOD (metastases to lung, soft tissue, bone, 2 mo)
		M	65	Retroperitoneum	N	14.8	/	Neoadjuvant radiotherapy Surgery	Malignant epithelioid to spindle cell neoplasm in a fascicular-herringbone pattern	/
		M	56	Left hand	N	4.5	1 yrs	Surgery	MIFS-like	AWOD (36 mo)
		M	48	Left knee	Y	5	/	Local radiotherapy and systemic chemotherapy	MIFS-like	AWD (5 mo)
		M	41	Left arm	Y	1.3	3–4 mo	Surgery	Malignant neoplasm of sheets and clusters of spindled to epithelioid/histiocytoid cells	AWOD (2 mo)
Huang et al. [56]	2023	F	9 mo	Liver	N	5 × 4 × 4.2	/	Surgery	EHE, Cavernous hemangioma	/
Jones et al. [57]	2023	F	55	Thigh	N	1.5	10 yrs	Surgery	RHE, EHE, Intraluminal papillary projections	AWOD (1.5 yrs)
Latypov et al. [58]	2023	M	61	Left kidney	N	5.0 × 6.0 × 4.0	/	Surgery	SCHE	RR (9 mo)
Schaeffer et al. [59]	2023	F	59	Heart	N	2 × 3	2 yrs	Surgery	EHE, RHE, SCHE	NSR or metastasis (24 mo)
Cordier et al. [60]	2024	M	41	Back of head; occipital skull bone	N	2	6 mo	Open biopsy	RHE, EHE	NSR or metastasis
Liu et al. [61]	2024	M	41	Intracranial	N	10.3 × 4.8 × 4	2	Surgery, radiotherapy	EHE, RHE, Hemangioma	/
Linos et al. [62]	2024.	F	35	Brachial plexus	N	4.8 × 2.6 × 3.7 (radiologic)	/	Surgery	RHE, EHE	NSR (4 mo)
		M	24	Plantar foot	N	≥5	/	Surgery	RHE, EHE	NSR (4 mo)
		F	80	Medistinum	N	N/A	/	Surgery	RHE, Cavernous hemangioma	RR (40 mo), no evidence of disease
		M	38	Abdominal wall	N	1.5	/	Surgery	EHE, RHE	/
Mao et al. [63]	2024	M	36	Mandibula	N	3.1 × 2.6	10 mo	Surgery	SCHE-like, RHE-like, EHE-like	NSR (40 mo)
Panizzardi et al. [64]	2024	F	2	Left leg	N	3	At 5 mo of age	Surgery	RHE, Pseudopapillary formations	NSR through age 7 y
Present case	2024	M	61	Spleen and liver	Y	16 × 14.5 × 10	1 yrs	Surgery and liver biopsy	Cavernous HE, EHE, Spindle cell hemangioma	DOD (6 mo)

HE—hemangioendothelioma, EHE—epithelioidHE; SCHE—spindle cell HE, AS—angiosarcoma, RHE—retiform HE, AV—arteriovenous; KHE—Kaposiform HE, PILA—Papillary intralymphatic angioendothelioma; UPS—undifferentiated pleomorphic sarcoma; MIFS—myxoinflammatory fibroblastic sarcoma; N/A—not applicable; mo—month; * M, male; F, female; Y, yes; N, no; NSR, no sign of reccurence; RR, reccurence; yrs, years; AWOD, alive without disease; mo, months; AWD, alive with disease; DOD, dead of disease. º Clinical follow-up information was available for 8 of 11 patients (range 6–28 months, median 10 months). † Median of “within months”—6 months.

**Table 4:** Immunohistochemical results of cases included in the systematic review.

Author	Year	CD31	CD34	FVIII	ERG	S100	D2-40	Ki67 *	HHV8	Desmin	SYN	Other
Nayler at al. [1]	2000	pos	pos	pos	/	/	/	/	/	/	/	/
		pos	pos	pos	/	/	/	/	/	/	/	/
		pos	pos	pos	/	/	/	/	/	/	/	/
		pos	pos	pos	/	/	/	/	/	/	/	/
		/	/	/	/	/	/	/	/	/	/	/
		pos	pos	pos	/	/	/	/	/	/	/	/
		pos	pos	pos	/	/	/	/	/	/	/	/
		pos	pos	pos	/	/	/	/	/	/	/	/
Reis-Filho et al. [13]	2002	pos	pos	pos	/	/	/	/	/	/	/	pos: CD68 neg: actin, desmin
Sapunar et al. [14]	2003	/	/	/	/	/	/	/	/	/	/	/
Biagioli et al. [11]	2005	pos	neg	/	/	/	/	/	/	/	/	/
Chu et al. [15]	2006	pos	pos	pos	/	/	/	42%	/	/	/	pos: VEGF neg: CK, EMA
Tronnier et al. [16]	2006	pos	/	pos	/	/	/	/	/	/	/	neg: actin
Fasolis et al. [17]	2007	neg	pos	pos	/	/	/	/	/	/	/	/
Fukuanga et al. [18]	2007	pos	pos	pos	/	/	neg	/	/	/	/	/
		pos	pos	pos	/	/	neg	/	/	/	/	/
		pos	pos	pos	/	/	neg	/	/	/	/	/
		pos	pos	pos	/	/	neg	/	/	/	/	/
		pos	pos	pos	/	/	neg	/	/	/	/	/
Requena et al. [9]	2007	pos	pos	pos	/	/	/	50%	neg	/	/	pos: Prox-1
Tejera-Vaquerizo et al. [19]	2008	pos	neg	/	/	neg	/	/	/	/	/	neg: cytokeratin-7
Utas et al. [20]	2008	pos	pos	pos	/	/	/	/	/	/	/	/
Aydingoz et al. [21]	2009		pos	/	/	neg	/	/	/	neg	/	pos: CD68 neg: keratin
Cakir et al. [22]	2009	pos	pos	pos	/	/	/	15%	/	/	/	pos: VEGF, SMA
Tsai et al. [23]	2011	pos	pos	/	/	/	pos	/	neg	/	/	pos: FLI-1
		pos	pos	/	/	/	neg	/	neg	/	/	pos: FLI-1
		pos	pos	/	/	/	pos	/	neg	/	/	pos: FLI-1
		pos	pos	/	/	/	pos	/	neg	/	/	pos: FLI-1
Chen et al. [24]	2012	pos	pos	/	/	neg	/	/	/	/	/	neg: vimentin, CK-7
Yoda et al. [25]	2012	pos	/	/	/	/	/	/	/	/	/	pos: FLI-1
Liau et al. [26]	2013	pos	/	/	/	/	/	/	/	/	/	pos: FLI-1 neg: AE1/AE3
McNab et al. [27]	2013	pos	pos	/	/	/	/	40%	neg	/	/	pos: CK8/18, AE1/AE3 neg: EMA
Tateishi et al. [28]	2013	pos	pos	pos	/	/	pos	/	/	/	/	pos: VEGF neg: cytokeratin CAM 5.2
Zhang et al. [29]	2013	pos	pos	pos	/	neg	/	/	/	/	/	neg: SMA, cytokeratin, EMA
Dong et al. [10]	2014	/	/	/	/	/	/	/	/	/	/	/
Mahmoudizad et al. [5]	2014	pos	neg	pos	/	neg	pos (rare)	20%	neg	neg	/	pos: vimentin neg: α-actin, KP1, AE1/3, factor XIIIa
Stojsic et al. [30]	2014	pos	pos	pos	/	/	neg	21%	/	/	/	neg: GLUT1
Leen et al. [31]	2015	pos	pos	/	pos	/	pos	/	neg	/	/	neg: AE1/AE3
Bhat et al. [32]	2016	/	pos	/	/	/	/	/	/	/	/	/
Perry et al. [4]	2017	pos	neg	/	pos	/	neg	/	/	/	pos	pos: FLI-1, CD56 neg: CGA, CK, CAMTA1
		pos	pos	/	pos	/	neg	/	/	/	pos	pos: FLI-1 neg: CGA, CD56, CK
		pos	neg	/	pos	/	pos	/	/	/	pos	pos: FLI-1, CD56 neg: CGA, CK, CAMTA
		pos	pos	/	pos	/	pos	/	/	/	pos	pos: FLI-1 neg: CD56, CGA, CK, AMTA1 (neg)
		pos	neg	/	pos	/	neg	/	/	/	pos	pos: FLI-1, CGA, CD56 neg: CK, CAMTA1
		pos	pos	/	pos	/	pos	/	/	/	pos	pos: FLI-1, CD56 neg: CGA, CK, CAMTA1
		pos	neg	/	pos	/	pos	/	/	/	pos	pos: FLI-1 neg: CGA, CD56, CK
		/	/	/	/	/	/	/	/	/	pos	neg: CGA, CD56, CK
		pos	pos	/	/	/	pos	/	/	/	pos	neg: CGA, CD56, CK
		pos	pos	/	pos	/	pos	/	/	/	pos	pos: FLI-1, CD56 neg: CGA, CK
		pos	pos	/	pos	/	pos	/	/	/	pos	pos: FLI-1 neg: CD56, CGA, CK
Rokni et al. [33]	2017	pos	pos	/	/	/	/	8%	/	/	/	neg: MNF116, SMA
Sakamoto et al. [34]	2017	pos	/	/	/	/	/	/	/	/	/	/
Umar et al. [35]	2017	/	pos	/	/	/	/	/	/	/	/	pos: vimentin neg: EMA
Harrington et al. [36]	2018	/	/	/	/	/	/	/	/	/	/	/
Antonescu et al. [8]	2020	/	/	/	/	/	pos	/	/	/	/	/
		/	/	/	/	/		/	/	/	/	/
		/	/	/	/	/	pos	/	/	/	neg	/
		/	/	/	/	/	/	/	/	/	/	/
		/	/	/	/	/	/	/	/	/	/	/
		/	/	/	/	/	/	/	/	/	/	/
		/	/	/	/	/	/	/	/	/	/	/
		/	/	/	/	/	/	/	/	/	/	/
		/	/	/	/	/	/	/	/	/	/	/
		/	/	/	/	/	/	/	/	/	/	/
		/	/	/	/	/	/	/	/	/	/	/
		pos	/	/	pos	/	/	/	/	/	pos	neg: chromogranin
Chin et al. [37]	2020	pos	pos	/	/	/	/	10%	/	pos	/	/
Cheuk et al. [38]	2020	pos	pos	/	pos	neg	pos	/	/	/	pos	neg: chromogranin, CD56, GFAP, AE1/3, MNF116, EMA, melan A, HMB45, CAMTA1, TFE3, FOSB
Gok et al. [39]	2020	pos	pos	/	/	/	/	10%	neg	neg	/	pos: thrombomodulin, podoplanin, CK7 neg: EMA
Langguth et al. [40]	2020	/	/	/	/	/	/	/	/	/	/	/
Li et al. [41]	2020	pos	pos	pos	pos	/	/	20%	/	/	/	pos: FLI-2 neg: AE1/AE3, TFE-3
Liao et al. [42]	2020	pos	neg	/	pos	/	neg	<1%	neg	/	/	/
Mani et al. [43]	2020	pos	/	/	pos	/	/	/	neg	/	/	/
		pos	/	/	pos	neg	/	/	/	neg	/	neg: pankeratin
Asilian et al. [44]	2021	/	/	/	/	/	/	/	/	/	/	/
Miyamoto et al. [45]	2021	pos	/	/	/	/	/	/	/	/	pos	neg: CD56, chromogranin
Dermawan et al. [46]	2021	pos	neg	/	pos	/	/	/	/	/	pos	neg: AE1/AE3, CD117, p63, calponin, INSM1
		pos	/	/	/	/	/	5%	/	/	pos	neg: pancytokeratin
Han et al. [47]	2022	pos	pos	pos	pos	/	/	3%	neg	/	/	/
Koutlas et al. [48]	2021	pos	pos	pos	pos	/	pos	2%	/	/	neg	pos: SMA, type IV collagen, CD68 neg: CAMTA1
Nakamura et al. [49]	2022	pos	pos	/	pos	/	neg	16.1%	/	/	/	pos: myo1B, CD146, SMA neg: CAMTA1, TFE3, FOSB
Zhang et al. [50]	2022	/	pos	/	pos	/	/	pos	/	/	/	/
Zhou et al. [51]	2022	pos	/	/	pos	/	pos	/	/	/	/	pos: Fli-1
Balko et al. [52]	2023	pos	neg	/	/	/	neg	50%	/	/	/	/
Bui et al. [53]	2023	pos	pos	/	pos	/	/	/	neg	/	pos	pos: FLI-1, MYC, neg: TFE3, CAMTA-1
Deng et al. [54]	2023	pos	pos	/	pos	/	/	5%	/	neg	/	pos: SMA, INI-1 neg: EMA, CK, PGMI
		pos	pos	/	pos	/	/	10%	/	/	/	neg: AE1/AE3, TFE-3, CAM5.2
Dermawan et al. [55]	2023	neg	/	/	neg	neg	/	/	/	neg	/	pos: SMA, caldesmon, CK OSCAR (rare) neg: Pan-CK, EMA, CK7, SOX10, HMB45, MDM2, CDK4, CD163, CD3, CD20, CD45, CD117, DOG1, Pan-NTRK, MUC4, ALK, Mel-A, TFE3, INI1, BRG1 and BRM retained.
		/	neg	/	/	neg	/	/	/	neg	/	pos: PDGFRB 4+, vimentin neg: AE1/AE3, CAM5.2, CK5/6, CK7, CK20, EMA, PSA, Mart-1, p63, TTFneg1, SMA, Myogenin, WT1, MUM1, ALK (D5F3), NUT, Pan-NTRK, CD45, CD56, CD34, CD20, ERG, CD117, CD21, SF-1, SOX10 and BRAF VE1. INI1, BRG1 and H3K27me3 (retained)
		/	neg	/	/	neg	/	/	/	pos	/	pos: DOG1, loss of H3K27me3 neg:AE1/AE3, SMA, myogenin, SOX10, MB45, MDM2
		/	neg	/	neg	neg	pos	/	/	neg	/	pos: YAP1, AE1/AE3, SMA (focal), EMA (focal) neg: HMBneg45, melan-A, MITF, SOX10, MyoD1, INI-1 (retained)
		/	/	/	/	pos	/	/	/	/	/	pos: AE1/AE3, EMA, p63, ERG, SOX10, INI1 (retained)
Huang et al. [56]	2023	pos	pos	pos	/	/	/	/	/		/	pos: FLI-1
Jones et al. [57]	2023	pos	neg	/	pos	/	/	5%	neg	/	neg	pos: podoplanin, GLUT1 neg: c-MYC, WT1, SOX10, chromogranin A, INSM 1, CD56
Latypov et al. [58]	2023	pos	pos	/	pos	/	/	40%	/	/	/	/
Schaeffer et al. [59]	2023	pos	/	/	pos	/	/	20%	/	/	/	neg: calretinin, CD30, CD15, Pax5
Cordier et al. [60]	2024	pos	pos	/	pos	neg	neg	/	/	/	neg	pos: SMA neg: CAMTA1, TFE3, Fosb, cMyc, CK, AE1/3, SOX10, CD56, chromogranin A
Liu et al. [61]	2024	pos	/	/	pos	/	/	3.6%	/	/	/	/
Linos et al. [62]	2024.	pos	pos	/	pos	/	neg	<5%	/	/	neg	pos: SMA
		pos	/	/	pos	/	pos	/	neg	/	pos	/
		/	/	pos	pos	/	/	5%	/	/	/	pos: SMA neg: CAMTA1, TFE3
		pos	neg	/	pos	/	neg	40%	/	/	pos	pos: CKAE1/AE3
Mao et al. [63]	2024	pos	pos	/	/	/	(pos)	15%	/	/	/	pos: FLI-1
Panizzardi et al. [64]	2024	pos	pos	/	/	/	pos	6%	/	/	/	neg: GLUT1, EMA, CK, AE1/AE3, WT1
Present case	2024	pos	neg	/	pos	neg	/	/	/	/	/	pos: vimentin, αSMA, CD68, LCA neg: CD34, AE1/AE3, EMA

* Highest Ki67 index.

**Table 5:** Results of genetic testing of cases included in the systematic review.

Author	Year	Genetics
Perry et al. [4]	2017	/
		/
		PTBP1-MAML2 fusion
		EPC1-PHC2 fusion
		/
		/
		/
		/
		/
		/
Antonescu et al. [8]	2020	pos YAP1-MAML2 fusion
		pos YAP1-MAML2 fusion
		pos YAP1-MAML2 fusion
		neg YAP1-MAML2 fusion
		neg YAP1-MAML2 fusion
		neg YAP1-MAML2 fusion
		neg YAP1-MAML2 fusion
		neg YAP1-MAML2 fusion
		neg YAP1-MAML2 fusion
		neg YAP1-MAML2 fusion
		neg YAP1-MAML2 fusion
		PTBP1-MAML2 fusion
Mani et al. [43]	2020	neg CAMTA1-TFE3 fusion
		neg CAMTA1-TFE3 fusion
Dermawan et al. [46]	2021	PTBP1-MAML2 fusion
		PTBP1-MAML2 fusion
Koutlas et al. [48]	2021	YAP1-MAML2 fusion
Balko et al. [52]	2023	No gene fusion identified, including YAP1-MAML2
Bui et al. [53]	2023	No gene fusion identified
Deng et al. [54]	2023	/
		neg FOSB-associated gene translocations
Dermawan et al. [55]	2023	ARHGAP42::MAML2 fusion mRNA expression: MAML2 and VGLL3 expression levels not upregulated
		ENDOD1::MAML2 fusion mRNA expression: MAML2 and VGLL3 expression levels not upregulated.
		ARHGAP42::MAML2 fusion
		YAP1::MAML2 fusion mRNA expression: VGLL3 upregulated; MAML2 not upregulated.
		YAP1::MAML2 fusion mRNA expression: MAML2, VGLL3 and YAP1 not upregulated.
		YAP1::MAML2 fusion mRNA expression: MAML2 and VGLL3 expression levels not upregulated
Schaeffer et al. [59]	2023	neg WWTR1-CAMTA1 fusion
Cordier et al. [60]	2024	pos YAP1::FOXR1fusion
Linos et al. [62]	2024	HSPG2::FGFR1 fusion
		YAP1::FOXR1 fusion
		ACTB::MAML2 fusion
		ARID1B::MAML2 fusion TP53 mutation: p.Pro151Ser

## Data Availability

The original contributions presented in this study are included in the article. Further inquiries can be directed to the corresponding authors.
